# Is the Weight of the Newborn Puppy Related to Its Thermal Balance?

**DOI:** 10.3390/ani12243536

**Published:** 2022-12-14

**Authors:** Karina Lezama-García, Julio Martínez-Burnes, Míriam Marcet-Rius, Angelo Gazzano, Adriana Olmos-Hernández, Patricia Mora-Medina, Adriana Domínguez-Oliva, Alfredo M. F. Pereira, Ismael Hernández-Ávalos, Uri Baqueiro-Espinosa, Ana de Mira Geraldo, Alejandro Casas-Alvarado, Daniel Mota-Rojas

**Affiliations:** 1 PhD Program in Biological and Health Sciences, [Doctorado en Ciencias Biológicas y de la Salud], Universidad Autónoma Metropolitana, Mexico City 04960, Mexico; 2 Animal Health Group, Facultad de Medicina Veterinaria y Zootecnia, Universidad Autónoma de Tamaulipas, Victoria City 87000, Mexico; 3 Animal Behaviour and Welfare Department, IRSEA (Research Institute in Semiochemistry and Applied Ethology), Quartier Salignan, 84400 Apt, France; 4 Department of Veterinary Sciences, University of Pisa, 56124 Pisa, Italy; 5 Division of Biotechnology—Bioterio and Experimental Surgery, Instituto Nacional de Rehabilitación-Luis Guillermo Ibarra Ibarra (INR-LGII), Mexico City 14389, Mexico; 6 Facultad de Estudios Superiores Cuautitlán, Universidad Nacional Autónoma de México (UNAM), Cuautitlán Izcalli 54714, Mexico; 7 Neurophysiology, Behavior and Animal Welfare Assessment, DPAA, Universidad Autónoma Metropolitana (UAM), Unidad Xochimilco, Calzada del Hueso 1100, Coapa, Col Villa Quietud, Coyoacán, Mexico City 04960, Mexico; 8 Mediterranean Institute for Agriculture, Environment and Development (MED), Institute for Advanced Studies and Research, Universidade de Évora, Pólo da Mitra, Ap. 94, 7006-554 Évora, Portugal; 9 School of Biological Sciences, Queen’s University Belfast, Belfast BT9 5DL, UK

**Keywords:** dogs, puppy welfare, animal perinatology, newborn puppy, thermoregulation, infrared thermography

## Abstract

**Simple Summary:**

Newborns experience a significant thermal change at birth, leading their bodies to adjust and reduce their metabolism to survive. In this study, newborn puppies’ weights and their temperatures in different body areas and at different measure times were evaluated to determine if there is a relationship between weight and their ability to reach thermostability. It was observed that there is a positive relationship between the weight of the puppies and their thermoregulatory capacity.

**Abstract:**

Hypothermia, a factor associated with neonatal mortality, can occur immediately after birth as a protective mechanism to prevent hypoxic damage in neonates, or to reduce the metabolic rate to improve the chances of survival in the first hours of life. The heat interchange through the superficial temperature of animals can be evaluated with infrared thermography (IRT). However, to date, there is no information on thermal windows in puppies. This study aimed to evaluate, with the use of IRT, the microcirculatory alterations in 8 different thermal windows identified at 7 different times in 289 newborn puppies assigned to different groups. Three thermograms were taken from four zones of each puppy: the facial, frontal, right lateral, and left lateral regions. Newborn puppies were grouped in 4 quartiles according to their weight: Q_1_ (126–226 g) *n* = 73, Q_2_ (227–330 g) *n* = 72, Q_3_ (331–387 g) *n* = 74, and Q_4_ (388–452 g) *n* = 70. A total of 8 thermal windows were considered at 7 evaluation times from Wet at birth until 24 h after birth (AB). Two-way mixed ANOVA within and between subjects’ design for each thermal window (eight models) was performed. Results revealed a positive correlation between the puppy’s weight and its ability to achieve thermostability in all the evaluated thermal windows. Statistically significant differences (*p* < 0.0001) between the 4 quartiles (Q_1_, Q_2_, Q_3_, and Q_4_) were found. The lowest temperatures were recorded when the pups were still wet and the highest at 24 h AB. Thermal windows with the highest temperatures were abdominal (34.234 ± 0.056 °C), thoracic (33.705 ± 0.049 °C), nasal (30.671 ± 0.110 °C), and upper left palpebral (34.066 ± 0.052 °C), while the lowest were thoracic limb brachial biceps (27.534 ± 0.051 °C), thoracic limb elbow (27.141 ± 0.049 °C), thoracic limb metacarpal (27.024 ± 0.062 °C), and femoral pelvic limb (27.654 ± 0.055 °C). Assessing the thermal response in newborn puppies can help identify drastic temperature reductions or deficient thermoregulatory compensation during the first hours of life, preventing the consequences of hypothermia.

## 1. Introduction

Endothermic animals regulate their body temperature through heat interchange between the amount produced by metabolism and the environment [1]. The control of heat exchange between the body surface and external environment plays a significant role in the regulation of body temperature during different physiological phases and/or activities throughout the life of homeotherms. Thermoregulatory adjustments can be induced not only by changes in environmental temperature but also by a variety of physiological situations including age, fasting and food intake, stress circumstances, and inflammation status, inducing changes in internal temperature which are followed by changes in body surface temperature. Under stressful conditions, animal’s reaction is mainly centered on the activation of the sympathetic system and the hypothalamic–hypophysis–adrenal axis (HPA) through the release of effector hormones, namely, catecholamine and glucocorticoid, respectively [2]. One of the known results of the sympathetic system’s activation is stress-induced hyperthermia, consisting in increased core body temperature with consequent changes in involucre temperature. Therefore, that allows obtaining distance images of a specific body region—represents a valuable tool to monitor the physiologic status, welfare, and stress responses of animals [3,4,5].

Thermoregulation plays an important role in newborns, especially in altricial species [6]. Through its extensive dermal vascularization, the body avoids losing temperature by producing changes in the blood flow of specific body regions [7], which are called biological thermal windows [8].

These changes can be indirectly quantified by evaluating tissue heat radiation through infrared thermography (IRT) [9]. Using a tool that can record these changes could help to identify states of hypothermia, a factor related to mortality and one of the most important causes of death in neonates [10,11].

Perinatal mortality in some species, such as pigs [12] and dogs [13,14], is one of the biggest concerns for producers and breeders since it can represent an average of one puppy out of ten live births dying before two months of age [15]. In addition, the immaturity of newborn puppies makes them highly vulnerable. Neonate thermoregulation involves biochemical, anatomical, physiological [16,17], and endocrine mechanisms to trigger respiratory and vascular changes and activate metabolism to produce energy [18]. It has been suggested that hypothermia occurs immediately after birth as a protective mechanism to prevent hypoxic damage in the neonate and reduce the metabolic rate to improve the survival of the newborn in the first hours [19,20,21]. Another important risk factor for neonatal mortality in diverse species, such as humans, pigs, and cattle, is low birth weight [22,23].

Similarly, in dogs, low birth weight neonates have a higher risk of death. More precisely, this risk is 12 times greater than in animals with normal birth weight [22]. In this species, studies have been carried out on the effect of the dam’s weight on the puppy’s weight at birth, litter size, vitality, and survival [13,24,25]. Likewise, the effect of the dam´s weight on the presentation of asphyxia and newborn hematological values have been evaluated [26]. According to Plavec et al. [27], puppies that experienced severe distress postpartum had significantly worse survival rates in the first week of their lives (*p* = 0.0113 and *p* = 0.0231, respectively). Attempts have been made to establish the most appropriate thermal regions or windows that provide better information on thermal changes in veterinary medicine [28]. Thus, thermal utility windows in dogs are the lacrimal caruncle, eye, ear, thorax flanks, appendicular area, and face [29,30,31]. However, there still needs to be more information on thermal windows in puppies. This could aid in our understanding of the process of thermoregulation in newborn dogs and, thus, help reduce the high mortality rates in this species.

This study aims to evaluate the microcirculatory changes in the different thermal windows of puppies assigned to different groups according to weight. The repercussions of their presentation of hypothermia were also analyzed. Our hypothesis assumed that (1) bigger newborns will have fewer thermoregulation problems than those of small sizes; (2) the highest temperatures will be obtained in thermal windows closest to the brain and vital organs (palpebral, thoracic, abdominal, or nasal); and (3) the lowest temperatures will be the ones recorded in thermal windows furthest from vital structures (thoracic limbs and pelvic limbs). 

## 2. Materials and Methods

### 2.1. Facilities

The study was carried out in 10 veterinary clinics in the central zone of the municipality of Campeche, Campeche state, Mexico, a zone with a tropical climate and a temperature range between 36 and 40 °C. The participation of owners of pregnant bitches was requested. Bitches were monitored and offered medical attention to carry out prenatal stage control from day 25 of pregnancy to 48 h of the puppies’ life.

### 2.2. Study Population

In total, 289 puppies from 60 parturient bitches were recruited and divided into 4 groups using size-specific quartiles, following Mugnier et al. [32] and Tessi et al.’s [33] method. The first quartile (Q_1_) represents the lowest 25% of registered values, the second quartile (Q_2_) represents 25–50%, the third quartile (Q_3_) represents 50–75%, and the fourth quartile represents 75–100% (Q_4_). Animals in the group Q_1_ were considered low-weight, while those belonging to Q_4_, the highest 25% of registered values, were considered high-weight puppies. This classification is due to the great variety of sizes between the breeds of dogs—the greatest morphological variability within any land mammal species. In this sense, in dogs, we can find adult body weight ranges from 500 g in miniature breeds, such as Chihuahuas, to more than 100 kg in giant breeds, such as mastiffs [34]. Among the breeds included in this study were Chihuahua, German shepherd, Labrador, golden retriever, Great Dane, standard schnauzer, cocker spaniel, poodle, Scottish terrier, and Belgian shepherd. As a result of this considerable variation in body weights, birth weight should be analyzed according to breed size. Quartiles were calculated at the puppy level with this formula: Qa = Li ((aN/4 + Fi-1)/Fi) Ai, where Li is the lower limit of the class where the quartile is located, N is the sum of the absolute frequencies, Fi-1 is the accumulated frequency of the previous class, and Ai is the amplitude of the class, that is, the number of values contained in the interval. The groups were Q_1_ (126–226 g) *n* = 73 puppies, Q_2_ (227–330 g) *n* = 72 puppies, Q_3_ (331–387 g) *n* = 74 puppies, and Q_4_ (388–452 g) *n* = 70 puppies. 

The weight of the puppies was obtained using a digital scale from Salter Weight Tronix Ltd., West Bromwich, UK, as soon as the dam stopped licking and cleaning the amnionic fluids and placental membranes from them.

### 2.3. Infrared Thermography

**Figure 1:**
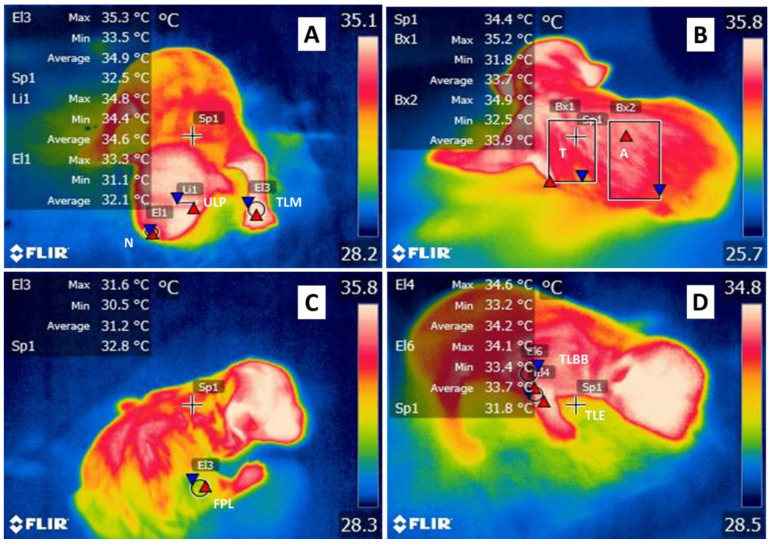
Thermal windows in newborn puppies. (**A**) Nasal (N) (El1), upper left palpebral (ULP) (Li1), and thoracic limb metacarpal (TLM) (El3) windows were made with circular figures delimited by the edges of the nasal mucosa, at the area of the edge of the left upper eyelid, and in the joint formed by the metacarpals covering the area from medial to lateral end, respectively. (**B**) Thoracic (T) window (Bx1) was made with rectangular figures delimited by the axillary area, the area of the last rib, and from the region of the spinal vertebrae to the ventral part of the abdomen. (**A**) Abdominal window (Bx2) was delimited by two millimeters after the last rib, to the inguinal area, and from the region of the spinal vertebrae to the ventral part of the abdomen. (**C**) Femoral pelvic limb (FPL) window (El3) was delimited by the space bounded by the edge of the pelvic limb in the biceps femoris region. (**D**) Thoracic limb brachial biceps (TLBB) (El6) and thoracic limb elbow (TLE) windows (El4) were obtained by placing circular figures from the area where the armpit begins to half the width of the thoracic limb and placing circular figures in the elbow area covering the vertex formed by the humerus-radio-ulnar joint, respectively.

In the present study, in each puppy, 8 thermal windows were identified at 7 different times, according to the methodology used by Lezama-García et al. [35]. The assessment times were: Wet (after the mother stopped licking the amniotic fluids from the pup), Dry (after drying the puppies with a towel for one minute), Colostrum (immediately after colostrum intake and after the puppy’s release of the teat), 30 min after birth (AB), 1 h AB, 4 h AB, and 24 h AB. The thermal windows are explained in detail in Figure 1. A total of 16,240 records of the puppies’ minimum, maximum, and average surface temperature were obtained. These data resulted from 289 puppies, from which 3 thermograms were taken from 4 zones: facial, frontal, right lateral, and left lateral regions. The facial images included the following regions: the upper left palpebral (ULP) and nasal (N). Frontal included thoracic limb metacarpal (TLM). Right lateral recorded femoral pelvic limb (FPL), thoracic limb biceps brachial (TLBB), and thoracic limb elbow (TLE), while on the left side, thoracic (T) and abdominal temperatures were assessed. Thermographic images were obtained with an infrared camera, FLIR^®^ model Thermal TM E80, FLIR Systems, Wilsonville, OR, USA, with the following specifications: IR resolution 320 × 240 pixels, thermal sensitivity <0.045 °C, accuracy ±2 °C or 2% of reading in the ambient temperature of 10 °C to 35 °C, and image frequency of 60 Hz. All images were collected with an emissivity of 0.95 at a uniform distance of 30 cm. The thermographic images were saved in JPEG format to analyze them later using specialized FLIR Tools software^®^ (FLIR Systems, Wilsonville, OR USA). The different evaluation times registered the minimum, average, and maximum temperatures for every image.

### 2.4. Statistical Analysis

Two-way mixed ANOVA within and between subjects’ design for each thermal window (eight models) was performed. This analysis has 4 independent categories (the groups according to weight of subjects) and 7 factors that were related or dependent (within subjects), also known as repeated measures.

Analysis components:

- Within subjects’ factor: 7 levels or times: (1) Wet; (2) Dry; (3) Colostrum; (4) 30 min; (5) 1 h; (6) 4 h; (7) 24 h.
- Between subjects’ factor: quartiles (4 levels): (1) Quartile 1 (Q_1_): 126–226 g *n* = 73; (2) Quartile 2 (Q_2_): 227–330 g *n* = 72; (3) Quartile 3 (Q_3_): 331–387 g *n* = 74; (4) Quartile 4 (Q_4_): 388–452 g *n* = 70.
- Interaction between factors: groups.

### 2.5. Ethical Statement

All the animal owners were asked to grant informed consent to carry out the research. This project was approved by the Ph.D. Program in Biological and Health Science Academic Committee, with approval number CBS.114.19. The animals included in the present study were treated gently and were not touched or stressed, since infrared thermography is a non-invasive technique. All work was performed under lineaments of Mexico’s Official Norm NOM-062-ZOO-1999 on technical specifications for animal production, care, and ethical use in applied ethological studies [36]. 

## 3. Results

The means and standard errors of the eight thermal windows were analyzed between the four quartiles. Statistically significant differences (*p* < 0.0001) between the 4 quartiles (Q_1_, Q_2_, Q_3_, and Q_4_) were found. However, in general and in all thermal windows, quartiles Q_2_ and Q_3_ were similar. In the same way, the evidence indicates significant differences between the temperature means of thermal windows and the different puppies’ evaluation times (Wet, Dry, Colostrum, 30 min AB, 1 h AB, 4 h AB, and 24 h AB).

The highest temperatures were recorded in the Q4 group puppies and the lowest in the Q1 puppies in all evaluated thermal windows (Table 1, Table 2, Table 3, Table 4, Table 5, Table 6, Table 7 and Table 8).

Table 1, Table 2, Table 3, Table 4, Table 5, Table 6, Table 7 and Table 8 show the estimated marginal means ± standard error. “Bonferroni corrections were used to adjust *p* values for multiple comparisons” to avoid a type-I statistical error, and the normal distribution of data was assessed using visual inspection of histogram and Q-Q plots.

Table 2 shows the average temperatures of the nasal window (N); a significant statistical difference (*p* < 0.0001) between group Q_1_ and Q_4_, with a difference of 2.48 °C between Wet time in Q_1_ and 24 h AB on Q_4_, can be observed.

Table 3 shows the range between different times on the TLM window in newborns with different weights classified into four quartiles. Moreover, like in previous tables, the average temperature in this thermal window shows that puppies with lower weights (Q_1_) recorded low temperatures, and puppies with higher weights show the highest temperatures. It is important to note that the lowest temperature of all windows at Q_1_ Wet time was recorded in this thermal window.

In Table 5, it can be observed that there is a significant difference *(p* < 0.0001) between the four groups at different times. However, there are similarities between groups Q_2_ and Q_3_, a pattern repeated in almost all thermal windows evaluated in this study. Similarly, it can be observed that the highest temperatures recorded in this study occurred in this thermal window at 24 h AB.

Table 6 shows statistically significant differences between the lowest and highest quartiles; however, at 24 h AB, the 4 quartiles practically remain without significant differences, except for Q_1_.

Table 7 shows that when comparing the Q_1_ group with Q_4_ at 24 h AB, the difference between these groups was 4.78 °C, and when comparing the temperatures of wet puppies of the Q_1_ group with temperatures recorded in the Q_4_ group at 24 h AB, a difference of 3.96 °C was recorded, but similitudes were observed again between group Q_2_ y Q_3_.

Table 8 shows that statistically significant differences between Q_1_ and Q_4_ at different times were more evident than in the rest of the thermal windows, and these differences were observed between groups Q_2_ and Q_3_, unlike what was observed in the remainder of the tables.

Subsequently, the correlations between the puppy’s weight and their temperatures at different times and in the different thermal windows were evaluated using Spearman’s rank correlations. Table 9, Table 10, Table 11, Table 12, Table 13, Table 14, Table 15 and Table 16 report the existing correlations between the puppies’ weight and their temperature, where the weights of the four groups (Q_1_, Q_2_, Q_3_, and Q_4_) and their respective temperatures by time and thermal window indicate how the temperature tends to vary at different times and in different thermal windows. In almost all cases, there is a positive correlation with statistically significant differences (*p* < 0.0001) (Table 9, Table 10, Table 11, Table 12, Table 13, Table 14 and Table 15). These results mean that the higher the weight of the puppies, the higher temperatures recorded in them. Only one case was observed where the correlation was negative (Table 16) in the thermal window TLE at 24 h AB. In this case, as the puppies’ weight increased, their temperature decreased in the TLE window. The *r* values in all tables were between 0.146 and 0.651, except in Table 16 of the TLE thermal window, where *r* = −0.024 (*p* = 0.679) at 24 h AB. The highest correlations were observed in Table 15 TLBB window (Colostrum *r =* 0.624) and Table 11 TLM window (Colostrum *r* = 0.632).

## 4. Discussion

The present study assessed the superficial temperature in 289 newborn puppies born from 60 bitches with different weights at 8 anatomical regions or thermal windows. The puppies were evaluated at different times and assigned to four groups using size-specific quartiles (Q_1_, Q_2_, Q_3_, and Q_4_). According to the results, there was an association between puppies’ thermal response and their weight, with the highest temperature recorded in Q_4_ in all thermal windows at all evaluation times. Additionally, although the temperatures at Wet and Dry times differed between the four groups (Q_1_, Q_2_, Q_3_, and Q_4_), both (Wet and Dry times) had the lowest values in all groups. Interestingly, at 24 h AB, the puppies’ temperature in every thermal window was similar between the groups, showing a similar thermoregulatory capacity independent of their weight; however, it was still the lowest in group Q_1_ and highest in Q_4_. These results differed from those of another study carried out by this workgroup [35], where the groups were classified according to the dam’s weight, and it was found that in the groups with greater dams’ weight, the thermoregulation of the puppies stabilized at 24 h AB, showing positive correlations with higher weight of the mother, due to better thermoregulation of her pups.

The present results show the effect of puppies’ weight on their superficial temperature. Newborns with low weight at birth had lower temperature values for all thermal windows at every measured time, compared with the heaviest puppies. According to Harri et al. [37], birth weight is important for the survival of neonates, and it is one of the most important factors that influence temperature loss, since the body mass index (BMI) determines the capacity of thermoregulation in newborns, as with foxes. However, the litter size also significantly influences (*p* = 0.003) the weight of the newborns. In this sense, it is common to have a higher number of low-birth-weight puppies in large litters compared to small litters [25]. This effect can be seen in piglets, where large litters can have smaller piglets with higher mortality rates, due to hypothermia or trauma generated by the mother when they are too weak to move [38]. 

This thermoregulatory dependence on weight and time has also been reported in dog puppies that cannot stabilize their temperature rhythm for several days after birth, but their rectal temperatures become stable at six weeks AB [39]. It is important to consider that the temperature of the environment in which puppies live could have a favorable or unfavorable effect on their welfare [40]. However, in this study, the temperature of the environment was not controlled, but similar conditions (34–40 °C) and foam mats were maintained in the place of parturition to avoid temperature loss by convection. Schrank et al. [41] reported that birth weight could impact newborns’ vitality and the health. It also has been reported that dogs of small size tend to lose heat quickly because they have a greater surface-to-area volume ratios [42]; therefore, they require a greater heat production to maintain their thermostability [43]. This can also be attributed to the wide variability of canine breeds [39]. 

Significant differences have been observed throughout the times evaluated, from wet to 24 h AB, in the evaluated regions. When the fetuses are still in the uterus of the bitch, they maintain a temperature of 0.3 to 0.5 °C higher than the dam’s body temperature. However, at birth, the puppies experience a significant thermal change and loss of temperature through evaporation occurs, because they are born wet, due to the amniotic fluid [44]. This happens because the insulation and protection function that the coat provides is not efficient, since the amniotic fluid has a high thermal conductivity which generates greater hypothermia. In addition to this, other contributing factors include decreased body fat, age, and lack of acclimatization to the environment [45,46]. Therefore, increased body temperatures when passing from Wet to Dry time may be explained either by the decrease in heat loss through evaporation, or the decrease in convection favored by air currents. Another critical factor is rubbing puppies, which stimulates dermal vascular microcirculation of peripheral blood vessels (peripheral vasodilation), increasing microvascular hyperemia at the dermal level. 

Hypothermia was observed in newborn puppies of the present study at the first three evaluation times (Wet, Dry, and Colostrum), where the lowest superficial temperatures were recorded. 

The thermostability observed at 24 h AB in puppies from all groups could be attributed to the efficacy of the thermoregulatory response of newborns regardless of their initial weight, as shown in the values of IRT in all thermal windows, where the four groups, when compared to the temperature at wet, had a similar average increase. Several factors can influence this response, such as glycogen reserves, colostrum intake, increased digestion, the presence of the dam, and the members of the litter. The thoracic windows demonstrate an important fact: as other studies carried out with other species showed, the temperature tends to rise after the consumption of colostrum; in this study, we can see that immediately after the consumption of colostrum, the temperature tends to rise in the four quartiles in the T window.

As observed in the results presented, at 24 h AB all puppies reached thermostability after their coat was dried and following colostrum intake and heat transfer of the mother’s temperature by convection. According to Münnich and Küchenmeister [21], these thermoregulatory behaviors are important during newborns’ first days since puppies cannot maintain their body temperature when exposed to cold environments up to six days after birth. 

According to the results reported in this study, the most notable thermoregulatory changes in newborn puppies were low temperatures in the distal regions, that is, in the thermal windows of TLM and FPL, and high temperatures in thermal windows A, T, and ULP. This could be because the most important structures related to metabolism and vital functions are found in the thoracic, abdominal, and cranial regions [47,48]. The appendicular windows presented the lowest temperatures, which can be explained, in the case of newborn puppies, because they still do not present locomotion, as they are an altricial species, and their degree of neurological development, responsible for the mechanism of thermal modulation, is immature. Neonates rely on peripheral vasoconstriction and arteriovenous shunts at the skin level to reduce heat loss, but also use non-shivering thermogenesis by lipolysis of brown adipose tissue and the oxidation of fatty acids in the mitochondria through adenosine triphosphate (ATP) synthesis, because it is reported that neonatal skeletal muscles are immature at birth. It is precisely this point that provides the explanation for why thermogenesis by shivering in newborn puppies is not so effective [44]. The time the altricial species develop the ability to obtain thermostability also influences this. According to Pineda and Dooley [49], thermostability is not reached until day 18 of life, and autonomic thermoregulation is known to be wholly developed at the end of the fourth week [19].

Additional research that could be performed based on the present findings includes study of the influence of factors such as environmental temperature, size of litter, and quality of maternal care on the thermostability of newborn puppies during the first hours of life. It is important to mention that one of the main limitations of this study was that, since births did not always occur in the same place, the environmental temperatures could not be controlled and standardized for all cases. Likewise, applying IRT to study the association with the percentage of male puppies would be interesting, since it was found that the percentage of male puppies was positively correlated with lateral nursing, a position that provides puppies the easiest access to the milk. Looking at this data from an evolutionary point of view, the mothers spend more attention on male puppies because it is more convenient in terms of fitness [50] and could alter the peripheral thermal response of neonates.

## 5. Conclusions

Results suggest that, during the first hours of the life of newborn puppies, their thermoregulation mechanism is deficient in anatomical and metabolic factors that can be reduced by colostrum intake, maintaining an adequate body temperature, and their mechanisms to conserve or dissipate heat in the evaluated thermal windows.

These results also suggest a positive correlation between the puppy’s weight and its ability to achieve thermostability, which is continuously observed in all the evaluated thermal windows. The puppies born with lower weights (Q_1_, 126–226 g) presented the lowest temperatures, while puppies with higher weights (Q_4_, 388–452 g) presented the highest temperatures. 

Likewise, the time effect of the temperature recording showed relevant changes in them; the lowest temperatures were obtained when the puppies were still wet and the highest at 24 h AB. 

Regarding the body regions where temperatures were evaluated, the findings suggest that the thermal windows with the highest temperatures were A, T, N, and ULP, and those with the lowest temperatures were TLBB, TLE, TLM, and FPL. Assessing the thermal response in newborn puppies can help identify drastic temperature reductions or deficient thermoregulatory compensation during the first hours of life, preventing the consequences of hypothermia.

## Figures and Tables

**Table 1:** Average of the temperature of upper left palpebral (ULP) window at different times in newborn puppies classified into 4 groups according to weight.

Time	Q_1_ *n =* 73		Q_2_ *n* = 72		Q_3_ *n =* 74		Q_4_ *n =* 70	
Wet	30.800 ±	0.065 ^b,5^	30.830 ±	0.066 ^b,5^	30.836 ±	0.065 ^a,b,5^	31.083 ±	0.067 ^a,5^
Dry	31.621 ±	0.083 ^b,4^	31.637 ±	0.084 ^b,4^	31.740 ±	0.082 ^a,b,4^	32.015 ±	0.085 ^a,4^
Colostrum	31.964 ±	0.072 ^b,3^	32.073 ±	0.073 ^b,3^	32.186 ±	0.072 ^a,b,3^	32.401 ±	0.074 ^a,3^
30 min AB	32.929 ±	0.052 ^b,2^	32.905 ±	0.053 ^a,b,2^	33.158 ±	0.052 ^a.2^	33.264 ±	0.053 ^a,2^
1 h AB	32.901 ±	0.039 ^c,2^	32.958 ±	0.039 ^b,c,2^	33.092 ±	0.038 ^b,2^	33.322 ±	0.039 ^a,2^
4 h AB	33.025 ±	0.078 ^b,2^	33.065 ±	0.079 ^a,b,2^	33.330 ±	0.078 ^a,2^	33.436 ±	0.080 ^a,2^
24 h AB	33.600 ±	0.051 ^b,1^	33.723 ±	0.051 ^b,1^	33.923 ±	0.050 ^a,1^	34.066 ±	0.052 ^a,1^

(Two-way mixed ANOVA.) Notes: *n*, number of puppies; weight of puppies according to category (Q_1_, 126–226 g; Q_2_, 227–330 g; Q_3_, 331–387 g; Q_4_, 388–452 g); AB, after birth; least-squares mean ± standard error. ^a,b,c^: different superscripts among columns indicate significant temperature differences between puppy’s weight groups at the same time. ^1,2,3,4,5^: different numbers among rows indicate significant differences between times in the same puppy’s weight group.

**Table 2:** Average of the temperature of nasal (N) window at different times in newborn puppies classified into 4 groups according to its weight.

Time	Q_1_ *n* = 73		Q_2_ *n* = 72		Q_3_ *n =* 74		Q_4_ *n =* 70	
Wet	28.187 ±	0.064 ^c,4^	28.649 ±	0.064 ^b,4^	28.711 ±	0.063 ^b,3^	29.127 ±	0.065 ^a,3^
Dry	29.757 ±	0.059 ^c,1,2,3^	30.002 ±	0.060 ^b,1,3^	30.331 ±	0.059 ^a,1^	30.431 ±	0.060 ^a,1,2^
Colostrum	29.445 ±	0.076 ^c,2^	29.860 ±	0.077 ^a,b,2,3^	30.036 ±	0.076 ^a,2^	30.293 ±	0.078 ^a,1,2^
30 min AB	29.385 ±	0.084 ^b,2^	29.645 ±	0.085 ^a,b,2^	29.924 ±	0.084 ^a,2^	30.167 ±	0.086 ^a,1,2^
1 h AB	29.494 ±	0.144 ^b,1,2^	29.619 ±	0.145 ^a,b,2^	29.994 ±	0.143 ^a,1,2^	30.224 ±	0.148 ^a,1^
4 h AB	29.366 ±	0.146 ^b,2^	29.697 ±	0.147 ^a,b,3^	30.080 ±	0.145 ^a,1,2^	30.377 ±	0.149 ^a,1^
24 h AB	29.977 ±	0.107 ^b,c,1^	30.281 ±	0.108 ^a,1,3^	30.453 ±	0.107 ^a,1^	30.671 ±	0.110 ^a,1^

(Two-way mixed ANOVA.) Notes: *n*, number of puppies; weight of puppies according to category (Q_1_, 126–226 g; Q_2_, 227–330 g; Q_3_, 331–387 g; Q_4_, 388–452 g); AB, after birth; least-squares mean ± standard error. ^a,b,c^: different superscripts among columns indicate significant differences of temperature between puppy’s weight groups at the same time. ^1,2,3,4^: different numbers among rows indicate significant differences between times in the same puppy’s weight group.

**Table 3:** Average of the temperature of thoracic limb metacarpal (TLM) window at different times in newborn puppies classified into 4 groups according to weight.

Time	Q_1_ *n* = 73		Q_2_ *n =* 72		Q_3_ *n =* 74		Q_4_ *n =* 70	
Wet	27.024 ±	0.062 ^c,6^	27.222 ±	0.062 ^c,5^	27.482 ±	0.061 ^b,6^	27.743 ±	0.063 ^a,5^
Dry	29.858 ±	0.066 ^c,3,5^	30.155 ±	0.066 ^b,2,4^	30.281 ±	0.065 ^a,b,2,5^	30.446 ±	0.067 ^a,3^
Colostrum	29.028 ±	0.038 ^c,4^	29.255 ±	0.038 ^b,3^	29.344 ±	0.037 ^b,4^	29.742 ±	0.038 ^a,4^
30 min AB	29.582 ±	0.081 ^b,3^	29.536 ±	0.082 ^b,3^	29.886 ±	0.081 ^a,3^	30.186 ±	0.083 ^a,3^
1 h AB	30.294 ±	0.057 ^b,c,2^	30.452 ±	0.057 ^b,2^	30.644 ±	0.056 ^b,2^	30.940 ±	0.058 ^a,2^
4 h AB	30.239 ±	0.196 ^b,1,2^	30.430 ±	0.197 ^b,1,2^	30.664 ±	0.194 ^a,b,1,2^	31.177 ±	0.200 ^a,1,2^
24 h AB	30.778 ±	0.086 ^b,1^	30.971 ±	0.086 ^a,b,1^	31.271 ±	0.085 ^a,1^	31.543 ±	0.088 ^a,1^

(Two-way mixed ANOVA.) Notes: *n*, number of puppies; weight of puppies according to category (Q_1_, 126–226 g; Q_2_, 227–330 g; Q_3_, 331–387 g; Q_4_, 388–452 g); AB, after birth; least-squares mean ± standard error. ^a,b,c^: different superscripts among columns indicate significant temperature differences between puppy’s weight groups at the same time. ^1,2,3,4,5,6^: different numbers among rows indicate significant differences between times in the same puppy’s weight group.

**Table 4:** Average of the temperature of thoracic (T) window at different times in newborn puppies classified into 4 groups according to weight.

Time	Q_1_ *n =* 73		Q_2_ *n =* 72		Q_3_ *n =* 74		Q_4_ *n =* 70	
Wet	28.884 ±	0.160 ^a,6^	28.861 ±	0.161 ^a,7^	29.089 ±	0.159 ^a,5^	29.194 ±	0.163 ^a,6^
Dry	31.525 ±	0.042 ^c,5^	31.657 ±	0.042 ^b,c,6^	31.806 ±	0.042 ^a,b,4^	31.902 ±	0.043 ^a,5^
Colostrum	31.718 ±	0.056 ^c,4^	31.904 ±	0.057 ^b,c,5^	31.982 ±	0.056 ^a,b,4^	32.119 ±	0.057 ^a,4^
30 min AB	32.067 ±	0.040 ^c,3^	32.176 ±	0.040 ^c,4^	32.358 ±	0.040 ^b,3^	32.520 ±	0.041 ^a,3^
1 h AB	32.218 ±	0.050 ^c,3^	32.380 ±	0.050 ^b,c,3^	32.453 ±	0.049 ^a,b,3^	32.601 ±	0.051 ^a,3^
4 h AB	32.504 ±	0.058 ^c,2^	32.666 ±	0.059 ^b,c,2^	32.734 ±	0.058 ^a,b.2^	32.910 ±	0.059 ^a,2^
24 h AB	33.251 ±	0.048 ^c,1^	33.451 ±	0.048 ^b,1^	33.596 ±	0.048 ^a,b,1^	33.705 ±	0.049 ^a,1^

(Two-way mixed ANOVA.) Notes: *n*, number of puppies; weight of puppies according to category (Q_1_, 126–226 g; Q_2_, 227–330 g; Q_3_, 331–387 g; Q_4_, 388–452 g); AB, after birth; least-squares mean ± standard error. ^a,b,c^: different superscripts among columns indicate significant temperature differences between puppy’s weight groups at the same time. ^1,2,3,4,5,6,7^: different numbers among rows indicate significant differences between times in the same puppy’s weight group.

**Table 5:** Average of the temperature of abdominal (A) window at different times in newborn puppies classified into 4 groups according to weight.

Time	Q_1_ *n* = 73		Q_2_ *n =* 72		Q_3_ *n =* 74		Q_4_ *n =* 70	
Wet	29.664 ±	0.100 ^a,5^	29.739 ±	0.101 ^a,5^	29.800 ±	0.099 ^a,5^	29.939 ±	0.102 ^a,5^
Dry	32.353 ±	0.039 ^c,4^	32.494 ±	0.039 ^b,c,4^	32.608 ±	0.038 ^b,4^	32.764 ±	0.039 ^a,4^
Colostrum	32.501 ±	0.063 ^b,4^	32.696 ±	0.063 ^a,b,4^	32.708 ±	0.062 ^a,b,4^	32.862 ±	0.064 ^a,4^
30 min AB	32.836 ±	0.062 ^b,3^	33.007 ±	0.063 ^a,b,3^	33.059 ±	0.062 ^a,b,3^	33.191 ±	0.064 ^a,3^
1 h AB	32.941 ±	0.069 ^b,3^	33.094 ±	0.069 ^a,b,3^	33.112 ±	0.068 ^a,b,3^	33.337 ±	0.070 ^a,3^
4 h AB	33.322 ±	0.071 ^b,2^	33.432 ±	0.072 ^a,b,2^	33.438 ±	0.071 ^a,b,2^	33.642 ±	0.073 ^a,2^
24 h AB	33.788 ±	0.055 ^c,1^	33.995 ±	0.055 ^b,1^	34.087 ±	0.055 ^a,b,1^	34.234 ±	0.056 ^a,1^

(Two-way mixed ANOVA.) Notes: *n*, number of puppies; weight of puppies according to category (Q_1_, 126–226 g; Q_2_, 227–330 g; Q_3_, 331–387 g; Q_4_, 388–452 g); AB, after birth; least-squares mean ± standard error. ^a,b,c^: different superscripts among columns indicate significant temperature differences between puppy’s weight groups at the same time. ^1,2,3,4,5^: different numbers among rows indicate significant differences between times in the same puppy’s weight group.

**Table 6:** The average temperature of the femoral pelvic limb (FPL) window at different times in newborn puppies classified into 4 groups according to weight.

Time	Q_1_ *n =* 73		Q_2_ *n =* 72		Q_3_ *n =* 74		Q_4_ *n =* 70	
Wet	27.654 ±	0.055 ^c,5^	27.979 ±	0.055 ^b,5^	28.001 ±	0.055 ^b,5^	28.348 ±	0.056 ^a,5^
Dry	28.670 ±	0.111 ^b,3^	28.826 ±	0.112 ^a,b,3,4^	29.072 ±	0.111 ^a,b,3,4^	29.116 ±	0.114 ^a,3^
Colostrum	28.020 ±	0.062 ^c,4^	28.280 ±	0.063 ^b,4^	28.515 ±	0.062 ^a,4^	28.714 ±	0.064 ^a,4^
30 min AB	28.363 ±	0.097 ^b,3,4^	28.535 ±	0.098 ^a,b,4^	28.611 ±	0.097 ^a,b,4^	28.846 ±	0.099 ^a,3,4^
1 h AB	28.732 ±	0.081 ^b,3^	29.072 ±	0.081 ^a,3^	29.137 ±	0.080 ^a,3^	29.366 ±	0.082 ^a,3^
4 h AB	29.815 ±	0.070 ^b,2^	30.068 ±	0.071 ^a,b,2^	30.226 ±	0.070 ^a,2^	30.434 ±	0.072 ^a,2^
24 h AB	30.825 ±	0.089 ^b,1^	31.163 ±	0.090 ^a,1^	31.186 ±	0.088 ^a,1^	31.483 ±	0.091 ^a,1^

(Two-way mixed ANOVA.) Notes: *n*, number of puppies; weight of puppies according to category (Q_1_, 126–226 g; Q_2_, 227–330 g; Q_3_, 331–387 g; Q_4_, 388–452 g); AB, after birth; least-squares mean ± standard error. ^a,b,c^: different superscripts among columns indicate significant temperature differences between puppy’s weight groups at the same time. ^1,2,3,4,5^: different numbers among rows indicate significant differences between times in the same puppy’s weight group.

**Table 7:** Average of the temperature of thoracic limb biceps brachial (TLBB) window at different times in newborn puppies classified into 4 groups according to weight.

Time	Q_1_ *n =* 73		Q_2_ *n =* 72		Q_3_ *n =* 74		Q_4_ *n =* 70	
Wet	27.534 ±	0.051 ^c,6^	27.840 ±	0.051 ^b,5^	27.883 ±	0.050 ^b,5^	28.344 ±	0.052 ^a,5^
Dry	28.439 ±	0.114 ^b,3,4^	28.615 ±	0.115 ^a,b,3^	28.948 ±	0.113 ^a,3^	29.043 ±	0.116 ^a,3^
Colostrum	27.884 ±	0.053 ^d,5^	28.127 ±	0.053 ^c,4^	28.440 ±	0.052 ^b,4^	28.693 ±	0.054 ^a,3,4^
30 min AB	28.206 ±	0.088 ^c,4^	28.399 ±	0.088 ^b,c,3,4^	28.543 ±	0.087 ^a,b,4^	28.839 ±	0.089 ^a,3,4^
1 h AB	28.630 ±	0.078 ^c,3^	28.988 ±	0.079 ^b,3^	29.111 ±	0.078 ^b,3^	29.421 ±	0.080 ^a,3^
4 h AB	29.567 ±	0.090 ^c,2^	29.876 ±	0.091 ^b,c,2^	30.149 ±	0.090 ^a,b,2^	30.329 ±	0.092 ^a,2^
24 h AB	30.712 ±	0.080 ^c,1^	31.023 ±	0.081 ^b,1^	31.024 ±	0.080 ^b,1^	31.500 ±	0.082 ^a,1^

(Two-way mixed ANOVA.) Notes: *n*, number of puppies; weight of puppies according to category (Q_1_, 126–226 g; Q_2_, 227–330 g; Q_3_, 331–387 g; Q_4_, 388–452 g); AB, after birth; least-squares mean ± standard error. ^a,b,c^: different superscripts among columns indicate significant temperature differences between puppy’s weight groups at the same time. ^1,2,3,4,5,6^: different numbers among rows indicate significant differences between times in the same puppy’s weight group.

**Table 8:** Average of the thoracic limb elbow (TLE) window temperature at different times in newborn puppies classified into 4 groups according to weight.

Time	Q_1_ *n =* 73		Q_2_ *n =* 72		Q_3_ *n =* 74		Q_4_ *n =* 70	
Wet	27.141 ±	0.049 ^d,7^	27.355 ±	0.049 ^c,7^	27.675 ±	0.049 ^b,5^	28.005 ±	0.050 ^a,5^
Dry	30.798 ±	0.062 ^b,6^	30.993 ±	0.062 ^b,6^	31.341 ±	0.062 ^a,4^	31.351 ±	0.063 ^a,3^
Colostrum	30.365 ±	0.066 ^b,5^	30.535 ±	0.067 ^b,5^	30.787 ±	0.066 ^a,3^	30.851 ±	0.068 ^a,4^
30 min AB	31.095 ±	0.069 ^c,4^	31.272 ±	0.069 ^b,c,4^	31.450 ±	0.068 ^a,b,4^	31.565 ±	0.070 ^a,2,3^
1 h AB	30.093 ±	0.061 ^c,3^	30.239 ±	0.062 ^c,3^	30.540 ±	0.061 ^b,3^	30.895 ±	0.062 ^a,2,4^
4 h AB	32.149 ±	0.069 ^b,2^	32.415 ±	0.070 ^a,2^	32.615 ±	0.069 ^a,2^	32.485 ±	0.071 ^a,1^
24 h AB	32.848 ±	0.081 ^a,b,1^	33.094 ±	0.082 ^b,1^	33.012 ±	0.081 ^b,1^	32.594 ±	0.083 ^a,1^

(Two-way mixed ANOVA.) Notes: *n*, number of puppies; weight of puppies according to category (Q_1_, 126–226 g; Q_2_, 227–330 g; Q_3_, 331–387 g; Q_4_, 388–452 g); AB, after birth; least-squares mean ± standard error. ^a,b,c,d^: different superscripts among columns indicate significant temperature differences between puppy’s weight groups at the same time. ^1,2,3,4,5,6,7^: different numbers among rows indicate significant differences between times in the same puppy’s weight group.

**Table 9:** Significant correlations between puppies’ weight and their superficial temperature in UPL thermal window.

Variables	Correlation Coefficient (*r*)	*p*-Value
Wet	0.190	0.001
Dry	0.189	0.001
Colostrum	0.248	<0.001
30 min AB	0.379	<0.001
1 h AB	0.444	<0.001
4 h AB	0.292	<0.001
24 h AB	0.364	<0.001

Spearman’s rank correlation coefficients and their statistical significance between puppies’ weight and their temperature at different times. AB: after birth.

**Table 10:** Significant correlations between puppy’s weight and their superficial temperature at N thermal window.

Variables	Correlation Coefficient (*r*)	*p*-Value
Wet	0.542	<0.001
Dry	0.510	<0.001
Colostrum	0.423	<0.001
30 min AB	0.365	<0.001
1 h AB	0.226	<0.001
4 h AB	0.353	<0.001
24 h AB	0.331	<0.001

Spearman’s rank correlation coefficients and their statistical significance between puppies’ weight and their temperature at different times. AB: after birth.

**Table 11:** Significant correlations between puppies’ weight and their superficial temperature at TLM thermal window.

Variables	Correlation Coefficient (*r*)	*p*-Value
Wet	0.551	<0.001
Dry	0.372	<0.001
Colostrum	0.632	<0.001
30 min AB	0.315	<0.001
1 h AB	0.469	<0.001
4 h AB	0.452	<0.001
24 h AB	0.353	<0.001

Spearman’s rank correlation coefficients and their statistical significance between puppies’ weight and their temperature at different times. AB: after birth.

**Table 12:** Significant correlations between puppies’ weight and their superficial temperature at T thermal window.

Variables	Correlation Coefficient (*r*)	*p*-Value
Wet	0.182	0.002
Dry	0.409	<0.001
Colostrum	0.307	<0.001
30 min AB	0.449	<0.001
1 h AB	0.337	<0.001
4 h AB	0.328	<0.001
24 h AB	0.441	<0.001

Spearman’s rank correlation coefficients and their statistical significance between puppies’ weight and their temperature at different times. AB: after birth.

**Table 13:** Significant correlations between puppies’ weight and their superficial temperature at A thermal window.

Variables	Correlation Coefficient (*r*)	*p*-Value
Wet	0.146	0.013
Dry	0.419	<0.001
Colostrum	0.258	<0.001
30 min AB	0.252	<0.001
1 h AB	0.273	<0.001
4 h AB	0.205	<0.001
24 h AB	0.376	<0.001

Spearman’s rank correlation coefficients and their statistical significance between puppies’ weight and their temperature at different times. AB: after birth.

**Table 14:** Significant correlations between puppies’ weight and their superficial temperature at FPL thermal window.

Variables	Correlation Coefficient (*r*)	*p*-Value
Wet	0.429	<0.001
Dry	0.415	<0.001
Colostrum	0.494	<0.001
30 min AB	0.224	<0.001
1 h AB	0.338	<0.001
4 h AB	0.365	<0.001
24 h AB	0.292	<0.001

Spearman’s rank correlation coefficients and their statistical significance between puppies’ weight and their temperature at different times. AB: after birth.

**Table 15:** Significant correlations between puppies’ weight and their superficial temperature at TLBB thermal window.

Variables	Correlation Coefficient (*r*)	*p*-Value
Wet	0.520	<0.001
Dry	0.444	<0.001
Colostrum	0.624	<0.001
30 min AB	0.311	<0.001
1 h AB	0.397	<0.001
4 h AB	0.406	<0.001
24 h AB	0.360	<0.001

Spearman’s rank correlation coefficients and their statistical significance between puppies’ weight and their temperature at different times. AB: after birth.

**Table 16:** Significant correlations between puppies’ weight and their superficial temperature at TLE thermal window.

Variables	Correlation Coefficient (*r*)	*p*-Value
Wet	0.651	<0.001
Dry	0.391	<0.001
Colostrum	0.429	<0.001
30 min AB	0.400	<0.001
1 h AB	0.525	<0.001
4 h AB	0.259	<0.001
24 h AB	−0.024	0.679

Spearman’s rank correlation coefficients and their statistical significance between puppies’ weight and their temperature at different times. AB: after birth.

## Data Availability

Not applicable.
